# Searching for Paralytic Toxin, Tetrodotoxin, in Swedish Bivalve Shellfish

**DOI:** 10.3390/md23060257

**Published:** 2025-06-19

**Authors:** Aida Zuberovic Muratovic, Shyamraj Dharavath, Jonas Bergquist, Malin Persson, Elin Renborg, Heidi Pekar, Mirjam Klijnstra

**Affiliations:** 1 Swedish Food Agency, P.O. Box 622, SE-751 26 Uppsala, Sweden; jadavraj@gmail.com (S.D.);; 2 Department of Chemistry, Biomedical Center, Analytical Chemistry and Neurochemistry, Uppsala University, P.O. Box 599, SE-751 24 Uppsala, Sweden; 3 Stockholm Vatten och Avfall, Bryggerivägen 10, SE-106 36 Stockholm, Sweden; 4 Wageningen Food Safety Research, Wageningen University and Research, Akkermaalsbos 2, 6708 Wageningen, The Netherlands

**Keywords:** tetrodotoxin (TTX), bivalve shellfish, screening, Sweden

## Abstract

Tetrodotoxin (TTX), earlier known as a tropical paralytic neurotoxin from pufferfish poisoning, has increasingly been occurring in edible marine species, including filter-feeding bivalves, from relatively cold marine waters of some European countries. The defined conditions that promote the production of TTX, its origin or the processes of its accumulation in seafood are still not clarified. Recent studies in temperate waters show, however, that the accumulation of quantifiable levels of TTX in bivalves appears to be influenced by seawater temperature (>15 °C), which indicates a seasonal occurrence at these latitudes. Uncertainties still remain regarding how seawater temperature interacts with other climate and environmental factors or organisms in the marine ecosystem to result in detectable levels of TTX in shellfish. Knowledge of the occurrence and distribution of TTX in the marine environment where the edible bivalves grow is important for maintaining seafood safety, as the toxin is heat-stable and remains potent even after cooking. Therefore, in this study, 264 bivalve samples collected in 2019 and 2021 from 17 sites along the Swedish west coast were analyzed with LC-MS/MS to search for TTX. The study explores the hypothesis of TTX presence in Swedish marine waters, outlines the sample screening strategy and objectives, and reports no evidence of TTX presence in Swedish bivalve shellfish (≥7.8 µg/kg) based on the analyzed samples and the time periods in which the studied samples were collected.

## 1. Introduction

Tetrodotoxin (TTX), is a heat-stable acute paralytic neurotoxin originally mostly known from poisonings caused by pufferfish (Tetraodontidae family) or local intoxications in Southeast Asia after consumption of seafood from areas associated with high-risk of TTX contamination [1,2,3]. TTX has been found not only in marine species but also in various terrestrial animals, as was earlier reviewed [4,5,6]. Yet, the consumption of edible marine species is the most common reason for human intoxication [1,7]. Following the ingestion of a TTX-contaminated meal, a clinical picture of a poisoning in humans can include a number of symptoms that appear from within 20 min up to 4 h [8,9]. The symptoms are a consequence of a non-functioning neurotransmission between the nerve cells, which might result in numbness (in the lips and mouth and can spread to the face and the extremities), gastrointestinal issues, slurred speech and muscle weakness that at higher doses progresses into paralysis, respiratory distress and sometimes fatality [7]. Although TTX became known as a pufferfish toxin, studies that later showed its absence in pufferfish specimens living in aquariums [10] or in other representative TTX-carrying organisms, like amphibians in captivity [11], raised the debate of the biogenic origin of TTX, including its biosynthesis that is yet to be elucidated [6,9]. The dynamics of TTX in ecosystems is complex and the toxin’s obvious presence in organisms has so far appeared to be based on an interplay between different factors, including surrounding habitat and environmental influences [9,12,13]. Also, the process of TTX accumulation has been complicated to clarify, as the sources of TTX presented in studies so far are multifaceted. At least in the case of marine organisms, and unlike other marine biotoxins, TTX has been primarily associated with production by bacterial species [14,15], including Vibrio, Bacillus, Pseudomonas and Actinobacteria [6,14,15,16,17,18], but also phytoplankton [19,20], the presence of nemertean species (ribbon worms) [12,21,22] and especially the links between the toxin occurrence, bacterial presence and climate change, with emphasis on elevated seawater temperatures [23,24,25,26].

In Europe, the TTX has been a non-issue until almost two decades ago when the first case of human poisoning was reported in 2007 from Spain after consumption of a gastropod *Charonia lampas* originating from Portugal [27,28,29]. This poisoning case, as evidence of emerging TTX in European marine waters, could likely correlate to the contemporary discovery on the presence of the pufferfish *Lagocephalus sceleratus* in the Mediterranean Sea reported in 2005, where its entrance (as a toxin-bearing species) was made possible through the Suez Canal [30,31,32]. The toxin spreading throughout the marine ecosystem and to other marine species indicates the involvement of TTX-producing microorganisms adapted to warmer water temperatures. The first detection of TTX in European bivalve molluscs was then reported in 2015 [16,19], which raised more concerns about seafood safety, resulting in a scientific opinion on the risks for public health related to the presence of TTX and its analogues in marine bivalves and gastropods (above a concentration of 44 µg/kg) from the European Food Safety Authority (EFSA) [33]. Since that, there has been growing evidence of TTX presence in edible marine species sourced from European marine waters as well as in other marine organisms sought as seafood [7,34,35,36]. However, there are no European regulatory requirements for monitoring the presence of TTX in bivalves yet. The responsibility for developing analytical methods for a reliable detection of TTX, as well as regular monitoring in seafood, remains a matter for each of the EU-member states.

So far, the threat from TTX has been deemed negligible in Scandinavian marine waters. Anyhow, there is evidence that TTX-contamination in seafood is spreading northwards. Several studies on the occurrence of TTX and its analogues in bivalve molluscs along the west-European coasts have been published during recent years, in northern France [26], The Netherlands [34], southern England [16,22,23,37], and Scotland (North Sea) being at the closest geographical distance to Sweden where the presence of TTX has been dated to 2014 [37]. Almost a decade later, it is reasonable to assume the presence of TTX in Swedish bivalve species, which raises concerns about health risks for consumers prompting the research on its discovery and handling.

The aim of this study was to search for the presence of TTX and its analogues in Swedish bivalve shellfish as a preventive measure in shellfish safety and, upon the discovery of TTX, respond to the call of EFSA on the need for more occurrence data from European waters in different EU Member States.

## 2. Results and Discussion

Proximity to the countries where TTX has been detected in shellfish over a decade ago and the different environmental conditions in Swedish marine waters, including the risk of bacterial production of TTX that increases with warmer climate, led to the hypothesis that TTX is present in Swedish bivalves at a detectable level. Given the complex origin of TTX and the interplay of various ecological factors in its emergence, conducting the present screening study was considered relevant to thereby confirm or reject this hypothesis at present.

**Figure 1:**
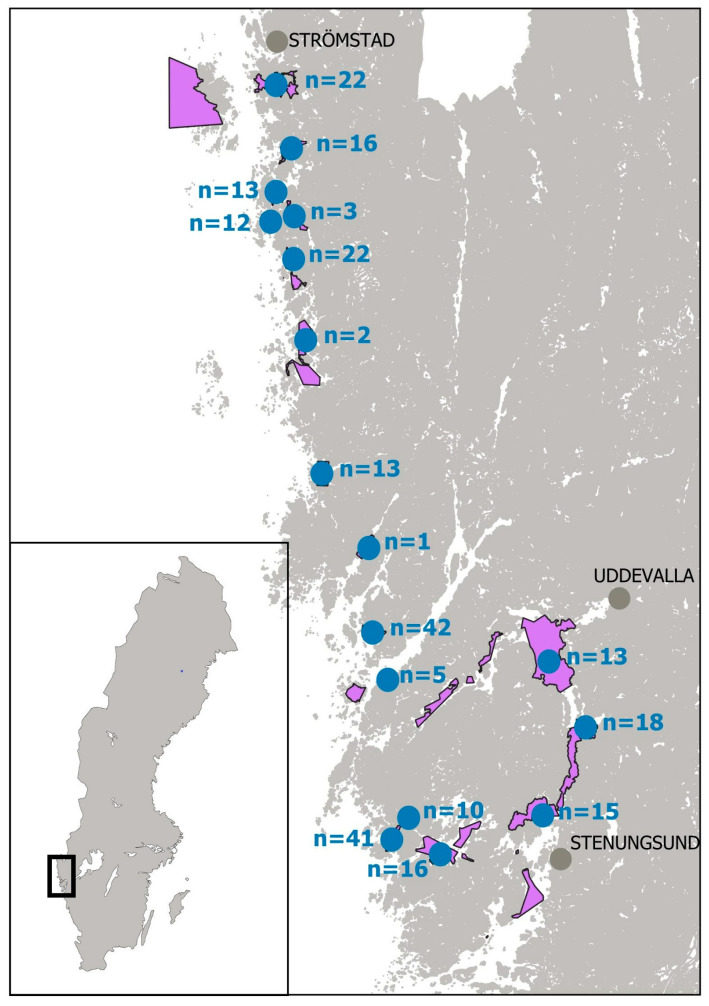
Map of Swedish bivalve molluscs production areas (purple) and selected sampling sites (blue). n = *n*, represents the number of samples per site.

Sampling sites for the study were selected, as far as possible, according to the risk profile proposed by Turner et al. [37]. The samples analyzed for TTX presence consisted of mussels (*Mytilus edulis*), Pacific oysters (*Magallana gigas*), native oysters (*Ostrea edulis*) and cockles (*Cerastoderma edule*). Most of these samples were official control (OC) samples from routine monitoring collected from classified production areas along the western Swedish archipelago (Bohuskusten) during the warmer months of April-September in 2019 and April-September in 2021, as shown in Figure 1. Sampling was limited to this part of the Swedish coastline, as it represents the only region in Sweden where production of live bivalve molluscs (LBM) occurs. Sampling beyond the summer season was not considered relevant since the occurrence of TTX in recent studies indicates a strong correlation to the temperatures above 15 °C.

**Figure 2:**
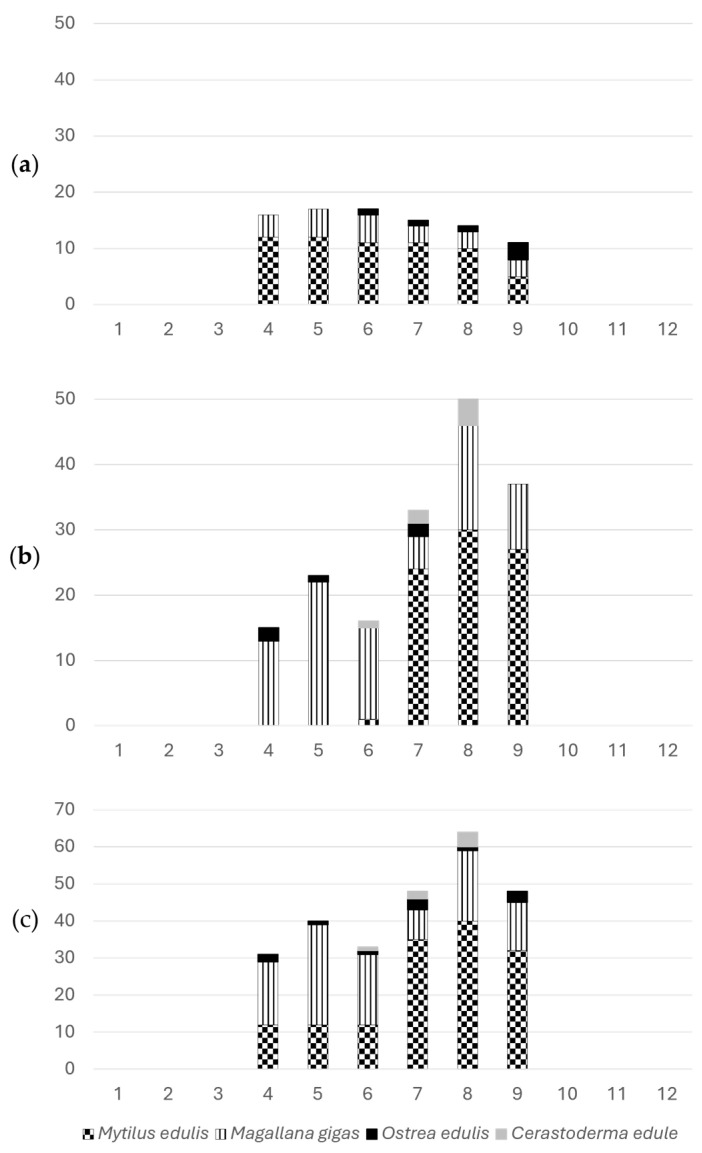
Number of samples tested in the study and their distribution across four species. (**a**) Number of samples per month 2019, (**b**) number of samples per month 2021, (**c**) total number of samples per month.

An additional five samples of *Magallana gigas* were collected from a selected area characterized by shallow water that tends to remain warmer for an extended period during the summer season, thereby creating conditions for potentially present TTX-producing microorganisms to prosper. When the presence of *Prorocentrum cordatum* was detected in the water column in September 2022, yet another group of mussel samples (*Mytilus edulis*) was collected, since earlier studies have assumed a linkage between the presence of *Prorocentrum* spp. and detectable levels of TTX [19,38]. Figure 2 shows the number of samples and proportion of representing species in each of the sampling seasons.

**Figure 3:**
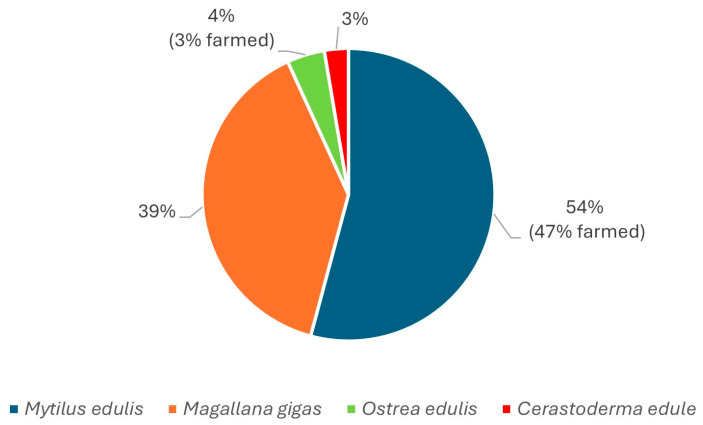
Proportion of each species in the study, of which farmed (or wild) per species.

The sampling strategy did not intend the collection of representative species for each monitoring site, rather, the goal was to broadly search across all species during the two warm seasons. Mussels (*Mytilus edulis*) is the slightly overrepresented species among the analyzed samples in Figure 3, which also reflects its dominance among the species harvested for human consumption in Sweden.

None of the tested samples were found to contain TTX, or the analogues for which the standards were available, above the limit of detection (LOD) for these toxins in the method, as shown in Table S1 [39]. Some samples that did not meet the specificity criteria for confirmation of TTX presence, yet exhibiting interesting signals (either through the presence of one of the two confirming fragment ions or a signal within the reasonable retention time window of a TTX-analogue that was included in the acquisition method although no standards were available) were sent to the national reference laboratory for marine biotoxins in The Netherlands for analysis using high-resolution mass spectrometry (HRMS). This approach could possibly clarify whether the signals could be from an isobaric toxin analogue to TTX with a similar elution time that could not be detected with the dictating triple quadrupole principle. The presence of TTX in these samples could not be confirmed with HRMS. Characteristic chromatograms of three representative samples and a calibration solution (at the limit of quantification, LOQ) of TTX, along with the calibration curve from the targeted analysis, are presented in (Supplementary Material Figures S1–S5), together with a summary of subsequent HRMS investigations.

As the purpose was primarily to conduct a first screening study of a potential presence of TTX in Swedish bivalves, no specific hydrographic parameters (salinity and depth) or seawater temperatures were recorded during sampling. Whether the absence of TTX in samples tested in this study is linked to a complex interplay of conditions unfavourable for its production at the time point of sampling at the respective sampling sites is therefore challenging to discuss. Factors influencing the temporal variability of TTX accumulation in bivalve molluscs appear to be difficult to reach a definitive conclusion about, based on data from previous European findings [13].

**Figure 4:**
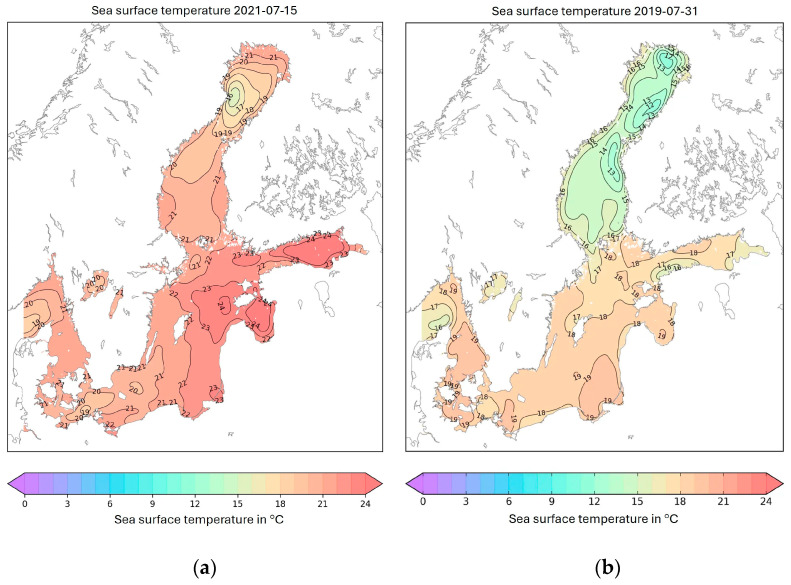
Sea surface temperatures in one day during each of the two summer seasons of samplings, (**a**) 15 July 2021, (**b**) 31 July 2019 [51,52].

However, several of the proposed sources of TTX, and the conditions in marine environments discussed in earlier studies as possible links or influencing factors that might interact in or trigger TTX production, are also present in Swedish marine waters:

1. The relationship between TTX and *Vibrio* bacteria is particularly interesting given the strong correlation between bacterial prevalence, TTX occurrence and warmer climate, as highlighted in previous research [14,40,41,42,43,44,45]. Coastal waters and brackish areas with moderate salinity, typically dominating in Scandinavian marine environments, favours *Vibrio* populations [46,47,48]. However, high abundance of *Vibrio* bacteria has also been reported in more saline waters of the Skagerrak Sea, especially in warm summer days [49]. One notable *Vibrio* outbreak in the Baltic Sea occurred during the warm summer in 2014, when the sea surface temperatures became unusually high [50]. This Vibrio outbreak temporally corresponded with a TTX-positive mussel sample (26 µg/kg) in Scottland (North Sea) [37], assumptively indicating the presence of TTX in Swedish marine waters (no samples were tested for TTX presence in Sweden during this period).
2. Furthermore, the assumption of TTX presence in Swedish marine environment is supported with the knowledge that sea surface temperature seems to be one of the ultimate criteria reported earlier for the occurrence of TTX [23,34,37]. The threshold of 15 °C was suggested to indicate a limit above which TTX is more likely to occur in shellfish. Certainly, surface temperatures of the seawater around Sweden can reach well above that threshold during the summer season, as exemplified for the sampling seasons of this study in Figure 4, and the summer of 2014 described above.

This notion could be further strengthened by the climate benefits from the Gulf Stream that, in combination with the climatic warming of coastal waters [53,54], interact and could create favourable conditions for the emergence of TTX even at these normally cold areas when compared to similar latitudes.

3. Introduction of invasive species, like the pufferfish or the spread of the invasive ribbon worm *Cephalotrix simula* to southern and northern Europe in the last decade, is strongly associated with the occurrence of TTX in European marine waters [21,55,56,57]. To the best of our knowledge, the latest biodiversity survey concerning nemerteans along the Swedish west coast was conducted in 2007 [58]. Various nemertean species have been studied in Sweden since then [59], but the occurrence of *C. simula* has not been reported yet. It is, however, reasonable to assume that the species could have become introduced into Swedish marine waters. One of the main vectors for the introduction of marine invasive species globally is ballast water of ships by which aquatic organisms are transported to new environments. Aspects of its constantly increasing impact on the existing ecosystems have been addressed elsewhere [60,61,62,63]. A majority of Sweden’s blue mussel production is located within 60 km north of the Port of Gothenburg, which is the largest port in Scandinavia.

With the above outlined conditions 1–3, one could suggest that there is no major difference in the general conditions for TTX occurrence in the marine environment compared to previous reports on TTX presence in northern Europe, which supports our hypothesis, while the results of this screening study could not confirm it.

## 3. Materials and Methods

### 3.1. Standards, Reagents and Chemicals

All the used standards, reagents and chemicals were as previously described by Patria et al. [39]. To summarize, certified reference material (CRM) of TTX and the analogues (in µmol/L concentrations 78.6, 9.9 of TTX and 4,9-anhydro TTX, and 2.50 of 11-deoxyTTX as non-certified, respectively) were obtained from Laboratorio CIFGA (Lugo, Spain). Stock standard solutions were prepared by diluting the CRM 10 times in Milli-Q^®^ water, from which the LC-MS/MS calibration standards were prepared in 80% acetonitrile with 0.25% acetic acid. Amorphous graphitized polymer carbon Supelco ENVI-Carb 250 mg/3 mL cartridges (Sigma-Aldrich, St. Louis, MO, USA) were used for clean-up of the raw shellfish sample extracts. Instrument solvents were LC-MS-grade where possible: acetonitrile (ACN, Fisher Scientific, Loughborough, UK), methanol and formic acid 98–100% (Merck, Darmstadt, Germany). Sample preparation reagents were of HPLC grade.

### 3.2. Sampling and Sample Preparation

Shellfish samples collected in warmer months were only considered relevant for screening purposes, as the risk profile described in previous studies from northern Europe showed that temperature plays a key role for TTX findings. In total, 264 bivalve samples (*Mytilus edulis*, *Magallana gigas*, *Ostrea edulis* and *Cerastoderma edule*), which according to the sampling procedure for regulatory monitoring of marine biotoxins all included at least 15 individuals, were collected within the period April–September in 2019 and 2021. All the 17 selected sampling sites shown in Figure 1 were marine areas along the Swedish west coast. After passing the official control of marine biotoxins monitoring in Sweden, samples selected for analysis in this study were stored, homogenized and frozen at −20 °C. Additional samples (*Mytilus edulis*) were collected in September 2022 from a site where *Prorocentrum cordatum* was present in the water column surrounding the bivalves. The number of samples tested per month of each season and the species represented in the sampling are shown in Figure 2. The number of samples per each of the 17 sampling sites, including the proportion (in %) of farmed or wild per species, are shown in Figure 3.

### 3.3. Screening Analysis

All samples were prepared, extracted and analyzed for the presence of TTX according to the method published by Patria et al. [39] without modifications, except that the multiple-reaction-monitoring (MRM) method comprised only the precursor and fragment ions of TTX and the analogues in the ion acquisition (Table S2). A matrix-matched calibration curve was analyzed, applying the bracketing calibration principle in each analysis batch. Samples resulting in non-specific, although interesting, signals within the expected retention time window were further investigated with HRMS to exclude or verify the possible presence of lesser known TTX analogues.

## 4. Conclusions

This study is the first to perform a large screening of a potential occurrence of TTX in bivalve molluscs collected along the coastal areas of the western Swedish archipelago.

The study was conducted using an in-house validated HILIC-MS/MS method [39], an analytical approach recommended by EFSA for the acquisition of occurrence data [33,36,64]. Based on the analysis of 264 samples, mostly oysters and mussels from 17 harvesting sites during two warm seasons in 2019 and 2021, this study provides no evidence of TTX presence in Swedish bivalve shellfish higher than the LOD of the applied method (≥7.8 µg/kg). As per these results, the risk to consumers of Swedish bivalves appeared to be negligible. Nevertheless, given that the results reflect the data from 2019 and 2021, they should be interpreted as a snapshot rather than a current assessment. The potential future presence of TTX in Swedish marine waters and bivalves should not be overlooked. Global warming and environmental changes may create favourable conditions for TTX production to prosper locally along a coast, thereby being represented in a low percentage of all bivalve shellfish, as previous studies have indicated [13,23,25].

Further research on Swedish bivalves is needed to increase the chance of detecting any possible presence of TTX and thereby prevent human intoxications, whether it would occur occasionally in specific locations described as “hotspots” [42,65,66], or at low concentrations. This is particularly important to highlight considering the occurrence of recreational shellfish harvesting during summers, as well as informal advice and trends to collect the easily accessible invasive oyster species *Magallana gigas* from shallow waters, thereby contributing to reducing its spread. Furthermore, the niche aquaculture in Sweden does not afford the research on their product safety, while the routine analysis using non-LC-MS/MS methods lacks the detection capability for TTX within the regulated monitoring of paralytic shellfish toxins (PSTs). A more effective strategy to discover a potential TTX-contamination in Swedish shellfish could be a continuous screening plan that should focus on the sites with the environmental features known to promote TTX accumulation (shallow water) during the warm season and with a higher sampling frequency (once–twice per week) over a period of several years.

## Data Availability

Data is contained within the article.

## Supplementary Materials

The following supporting information can be downloaded at: https://www.mdpi.com/article/10.3390/md23060257/s1, Table S1: LOD (limit of detection) and LOQ (limit of quantification) levels of toxins in the HILIC-MS/MS method applied in the screening study, Table S2: Settings of the LC-MS/MS parameters, Raw data chromatograms characteristic from targeted analysis with HILIC-MS/MS, Figures S1 and S2: calibration curve of TTX in blue mussel extract and chromatogram of the lowest level of calibration (LOQ) with TTX, respectively; Figures S3–S5: LC-HILIC MS/MS analyses of three different blue mussel samples showing presence of specific MRM fragments of 11-nor TTX-6-ol, 11-deoxyTTX/5-deoxyTTX and TTX, respectively. Summary of the subsequent HRMS analysis of the three samples, p5-17 (chromatograms and spectra of TTX standard and selected sample peaks).
