# Supplementary material for: Searching for Paralytic Toxin, Tetrodotoxin, in Swedish Bivalve Shellfish
Source: Mar Drugs. 2025 Jun 19;23(6):257. doi: 10.3390/md23060257 (PMC12194443; doi:10.3390/md23060257)
Supplement: Supplementary file 1 [file marinedrugs-23-00257-s001.zip › marinedrugs-3645474-supplementary.pdf]

# Supplementary Material:
# Searching for Paralytic Toxin, Tetrodotoxin, in Swedish Bivalve Shellfish

**Aida Zuberovic Muratovic 1,* , Shyamraj Dharavath 1, Jonas Bergquist 2 , Malin Persson 1, Elin Renborg 1, Heidi Pekar 3 and Mirjam Klijnstra 4**

[1] Swedish Food Agency, P.O. Box 622, SE-751 26 Uppsala, Sweden; jadavraj@gmail.com (S.D.)
[2] Department of Chemistry, Biomedical Center, Analytical Chemistry and Neurochemistry, Uppsala University, P.O. Box 599, SE-751 24 Uppsala, Sweden
[3] Stockholm Vatten och Avfall, Bryggerivägen 10, SE-106 36 Stockholm, Sweden
[4] Wageningen Food Safety Research, Wageningen University and Research, Akkermaalsbos 2, 6708 Wageningen, The Netherlands

**Table S1**. Validated LOQ and LOD levels of toxins (in shellfish matrix) in HILIC-MS/MS method applied in present screening study [39].

| Toxin | LOQ Conc. | LOD Conc. |
|---|---|---|
| | (µg TTX/kg) | |
| TTX, 4-epiTTX | 15.68 | 7.84 |
| 11-deoxyTTX | 1.89* | 0.95 |
| 4,9-anhydroTTX | 7.45* | 3.73 |

**Table S2**. Settings in the MRM method and the m/z transitions of TTX and its analogues using a Waters Xevo TQ-S triple quadrupole mass spectrometer [39]. Bold indicates primary MRM m/z transition for quantification of corresponding analogues. *Toxins for which no primary certified reference standard was available.

| Analytes | Precursor ion, m/z | Product ion, m/z | Cone, V | Dwell, ms | CE, eV | Mode |
|---|---|---|---|---|---|---|
| TTX, 4-epi TTX | **320.1** | **162.1**; 302.1 | 40 | 25 | 38; 25 | ESI+ |
| 11-deoxy TTX | **304.1** | 176.1; 286.1 | 40 | 25 | 30 | ESI+ |
| 4,9-Anhydro TTX | **302.1** | 162.1; 256.1 | 40 | 25 | 30 | ESI+ |
| *11-nor TTX-6-ol | 290.1 | 162.1; 272.1 | 40 | 25 | 30 | ESI+ |
| *5,6,11-Trideoxy TTX | 272.1 | 162.1; 254.1 | 40 | 25 | 30 | ESI+ |

Raw data from mass spectral experiments performed using HILIC-MS/MS on a Waters Xevo TQ-S triple quadrupole mass spectrometer in screening analysis for the presence of TTX in three representative mussel samples

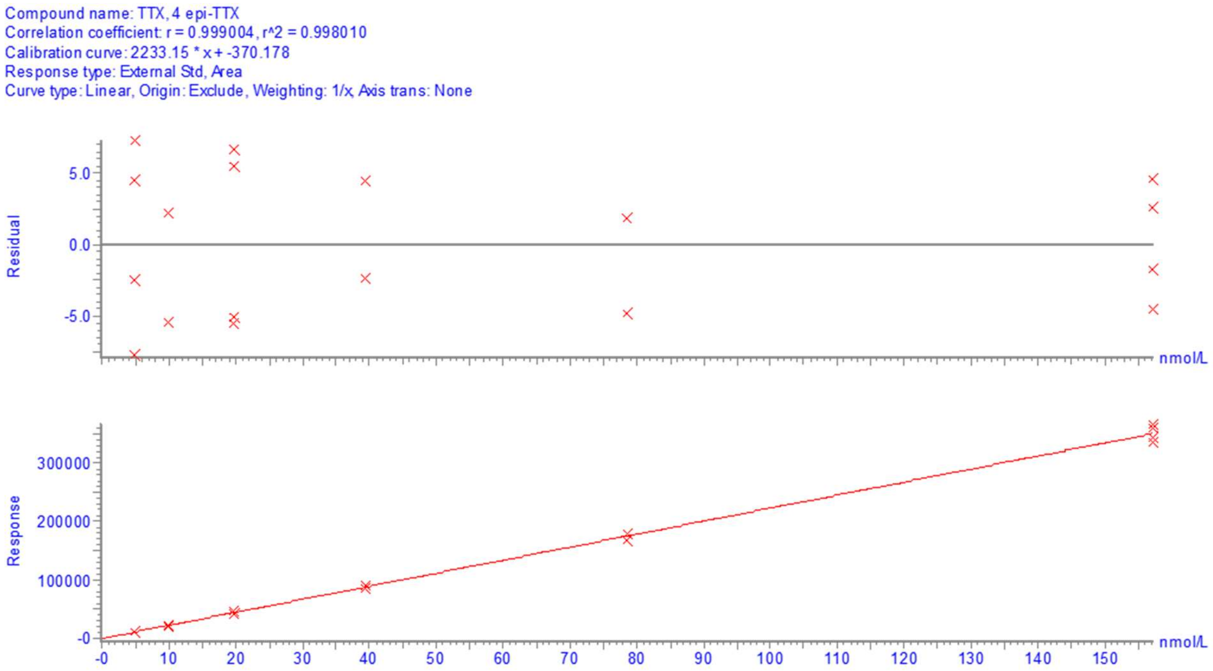


**Figure S1**. Calibration curve of TTX at 6 concentration levels in blue mussel (*Mytilus edulis*) matrix extract acquired using bracketing calibration principle within the same analysis batch as the sample chromatograms (20220211) shown in Figures S2-S5.

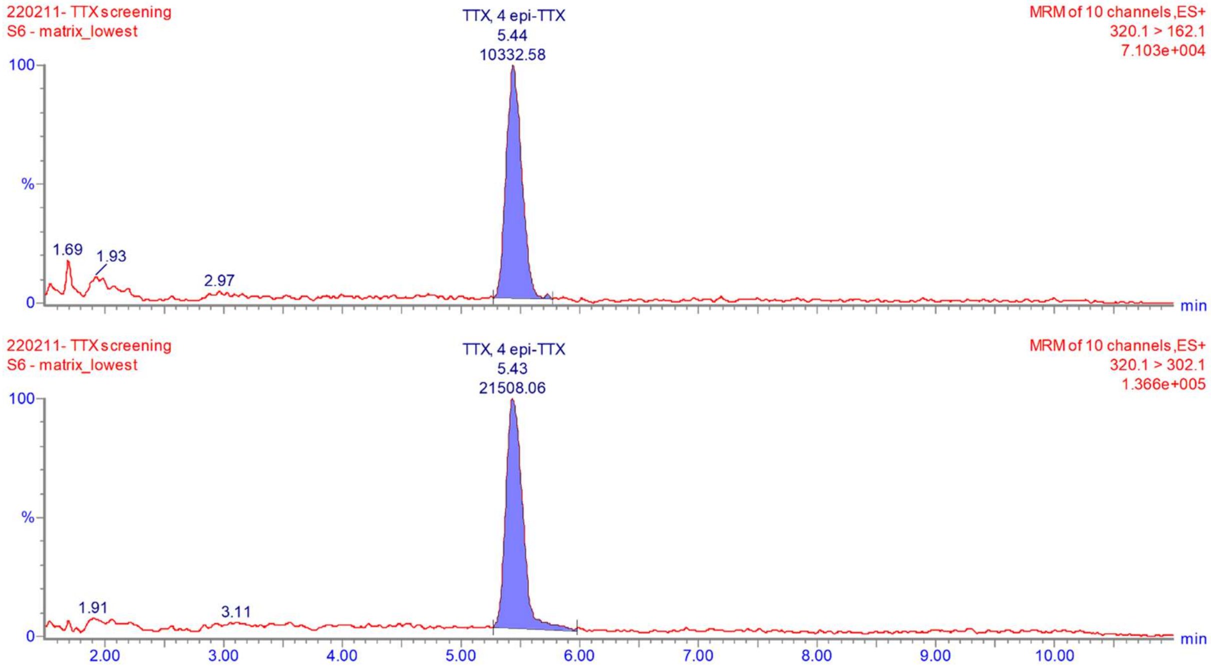


**Figure S2**. Lowest level of calibration in the method (LOQ) with TTX standard (CRM) in blue mussel (*Mytilus edulis*) matrix extract, bracketing calibration principle - batch start.

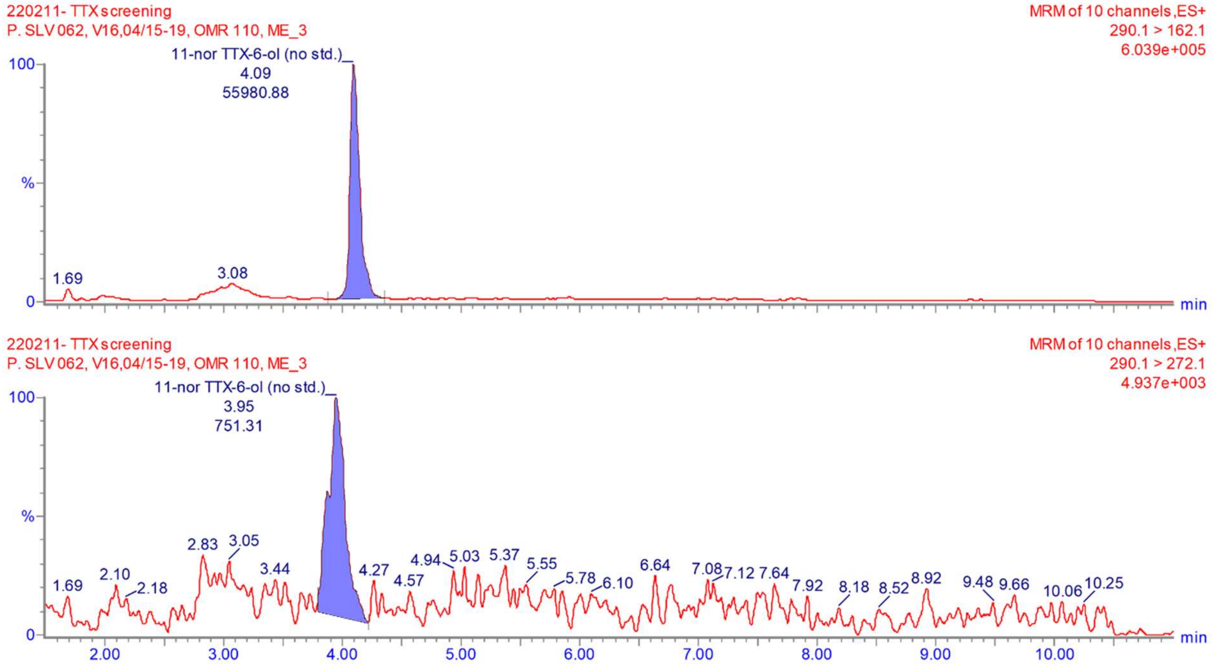


**Figure S3**. LC-HILIC MS/MS analysis showing presence of specific MRM fragments of the TTX-analogue, 11-nor TTX-6-ol, in a blue mussel (*Mytilus edulis*, ME) sample SLV062. The toxin presence could not be confirmed as no reference standard is available for this toxin. Sampling information: Week 16, April 15$^{th}$, 2019.

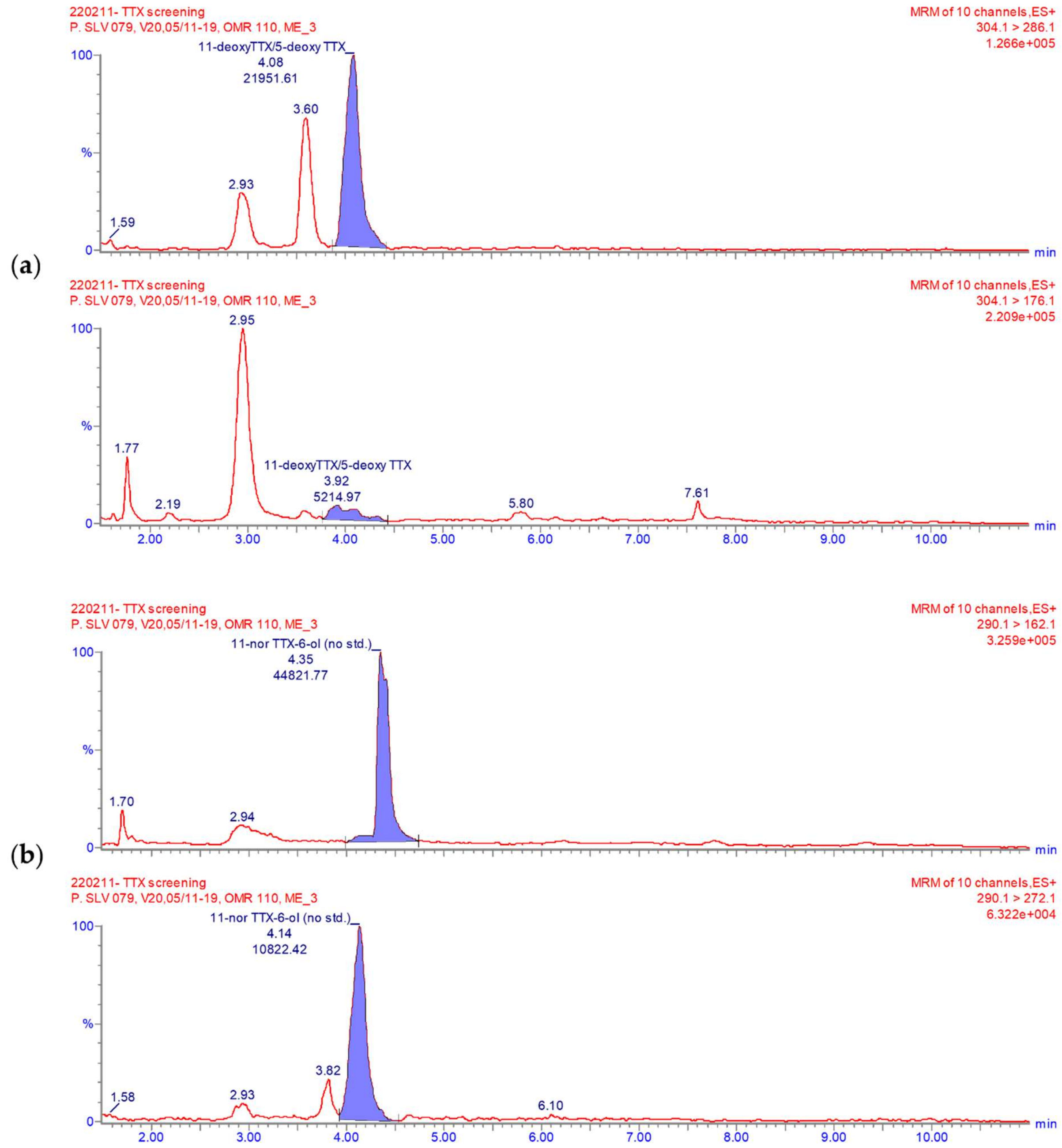


**Figure S4**. LC-HILIC MS/MS analysis showing presence of specific MRM fragments of the TTX-analogues **a)** 11-deoxyTTX / 5-deoxyTTX, and **b)** 11-nor TTX-6-ol, in a blue mussel (*Mytilus edulis*, ME) sample SLV079. The toxins presence could not be confirmed as no reference standards are available for these toxins. Sampling: Week 20, May 11th, 2019.

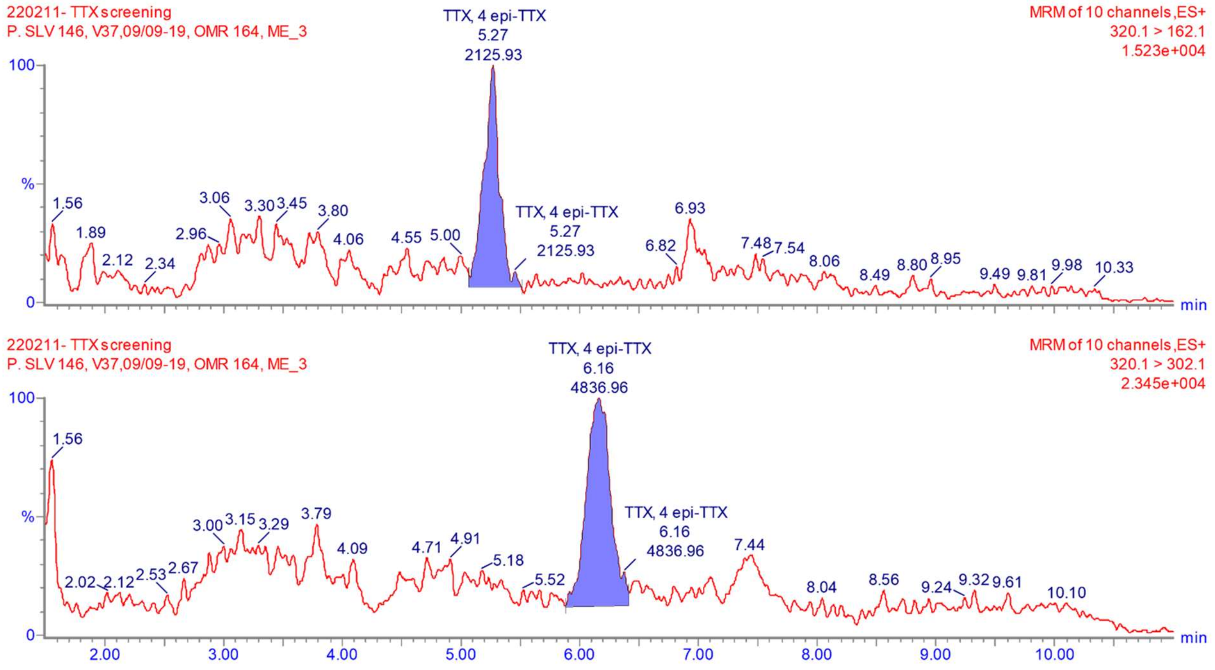


**Figure S5**. LC-HILIC MS/MS analysis showing presence of specific MRM fragments of TTX in a blue mussel (*Mytilus edulis*, ME) sample SLV146, although not aligned in retention time (Tr) and different compared to TTX-signal in calibration solution in Figure S2. Sampling: Week 37, September 9$^{th}$, 2019.

## Summary and the raw data of the subsequent HRMS investigations for the presence of TTX or its analogues in three representative mussel samples

### Sample preparation

2 g shellfish homogenate + 3 mL 1%Hac in MeOH. Single extraction, made up to 4 mL with water. Blank mussel extract was fortified with 125 µg/kg TTX.

### Chromatographic conditions

TSK gel Amide-80 2 µm, 150 · 3 mm
Gradient 90% ACN containing 20mM ammonium formate and 0.5% FA to water containing 100mM ammonium formate and 0.5% FA.

### MS conditions

SIM mode 320.1 and 290.1 with a 4 Da isolation window
Resolution: 70.000 FWHM
ACG target and maximum time were optimized in a way to gain as much signal as possible.

### Fragmentation

Targeted MS2 of 320.1 Da
Resolution 35.000 FWHM
HCD fragmentation with a NCE of 30

**Results**
Chromatograms below are each time:
- 4 Da window 320.1
- Exact mass TTX with a 5 ppm window
- 4 Da window 290.1
- Exact mass 11-nor TTX-6-ol with a 5 ppm window

**Spectra of all identified peaks**

**Conclusions**

Sample 1 (SLV062), 2 (SLV079) and 3 (SLV146) contain a peak with the exact mass of TTX (Δ ppm error max 2.13) at the retention time of TTX (6.23 min). Concentrations were too low to obtain fragmentation data and the TTX presence in the sample could not be fully confirmed.

Sample 2 (SLV079) also contains a peak with the exact mass of TTX (Δ ppm error 0.23) at a different retention time (5.60 min). The fragmentation pattern of the peak at 5.6 min does not fully correspond to that of TTX.

Sample 2 (SLV079) contains multiple peaks with the exact mass of 11-nor TTX-6-ol (Δ ppm error max 1.32). There is no standard available for this toxin, therefore the presence of 11-nor TTX-6-ol in the sample could not be confirmed.

There are many interferences present which causes that the identified peaks cannot be distinguished in the chromatogram of the 4 Da window (showing 4 Da window versus 5 ppm window allowed comparison of low-resolution data acquired with LC-MS/MS with high resolution data expected from HRMS).

**Standard 1: 125 µg/kg TTX at 6.23 min.**

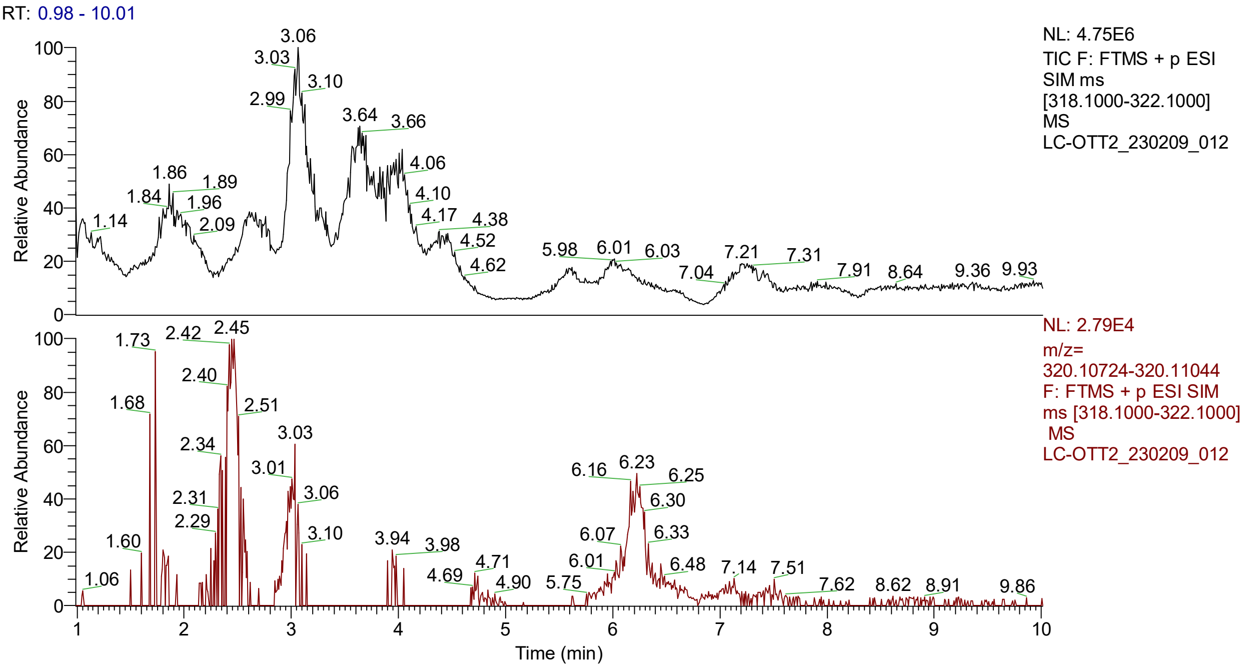


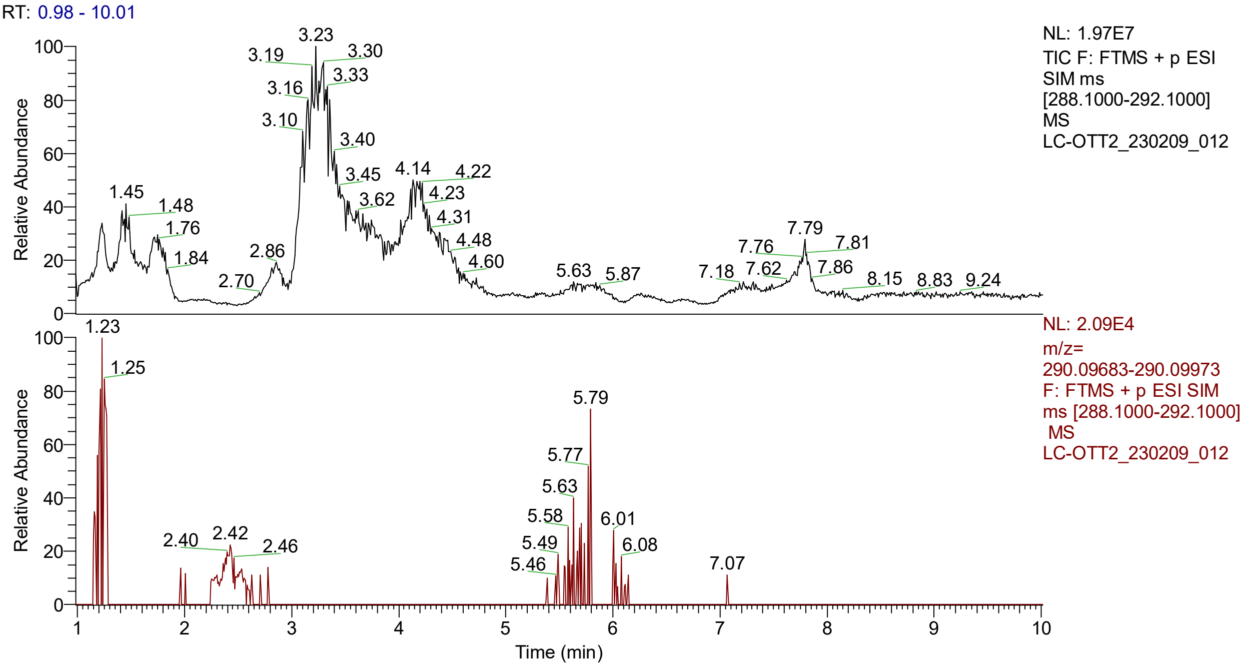

**Sample 1: SLV062 V16 04 / 15-19 OMR 110**

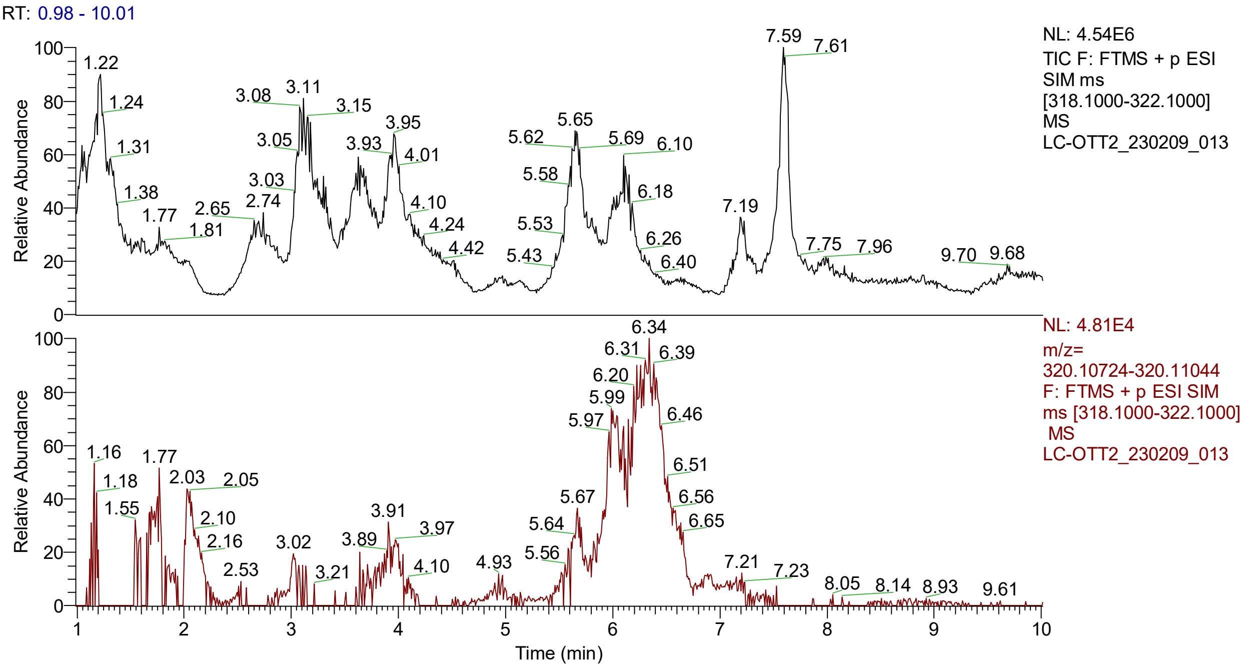


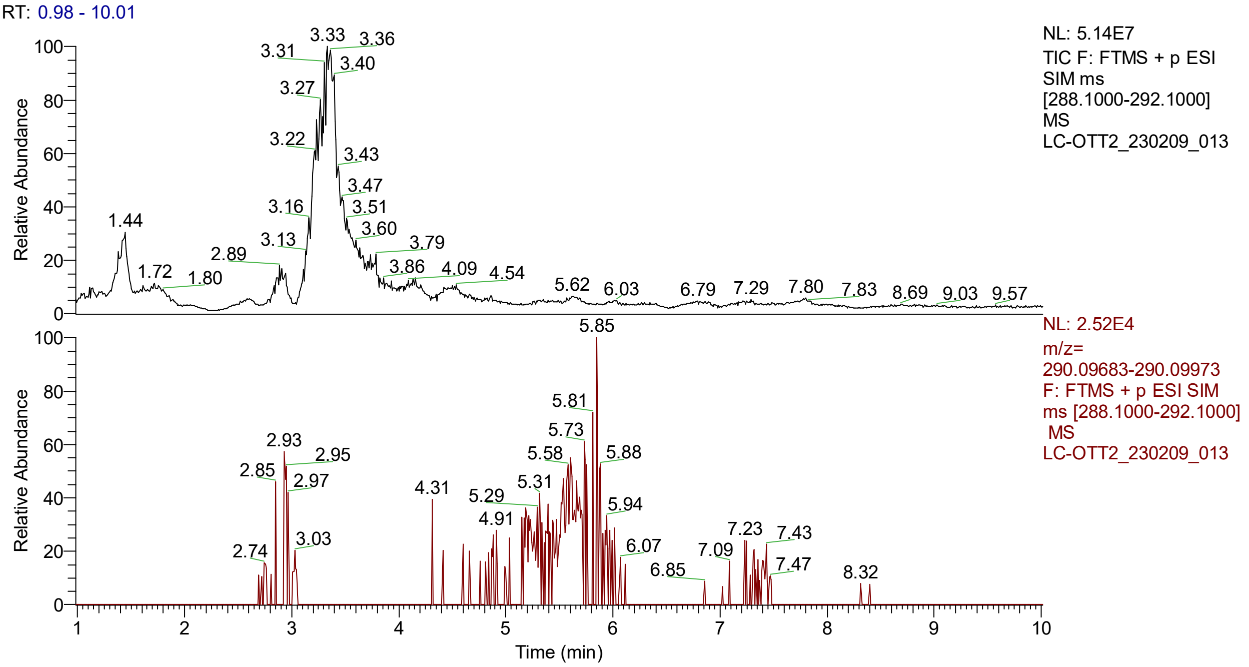

**Sample 2: SLV079 V20 05 / 11-19 OMR 110**

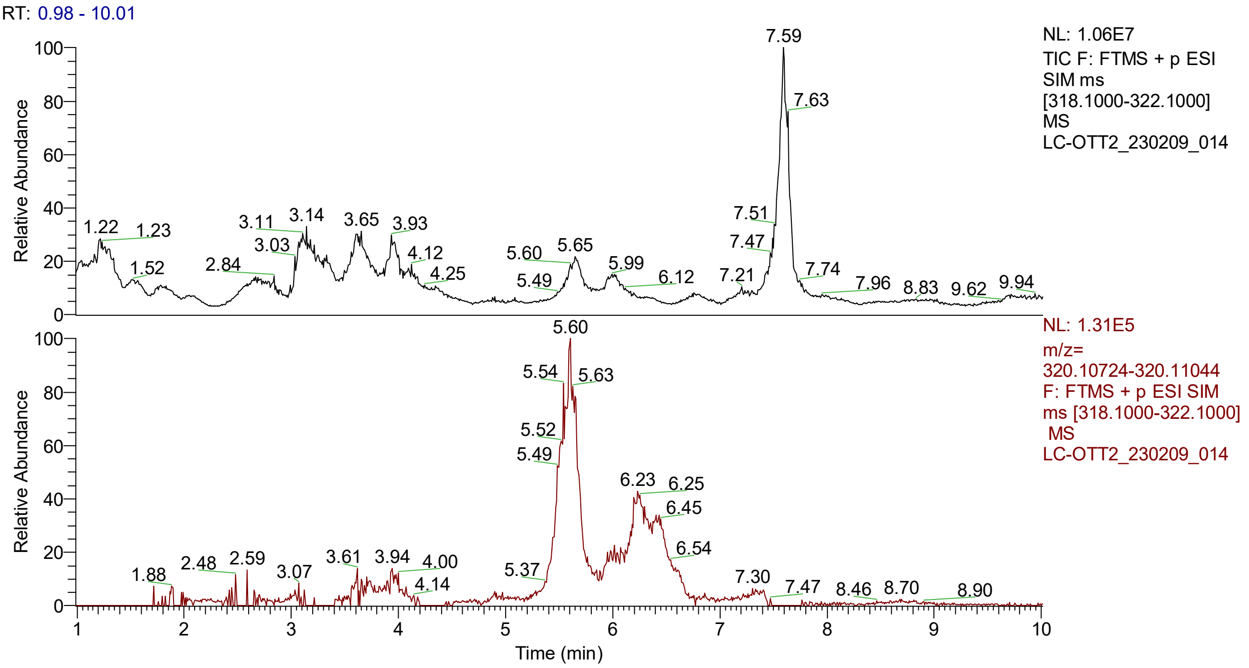


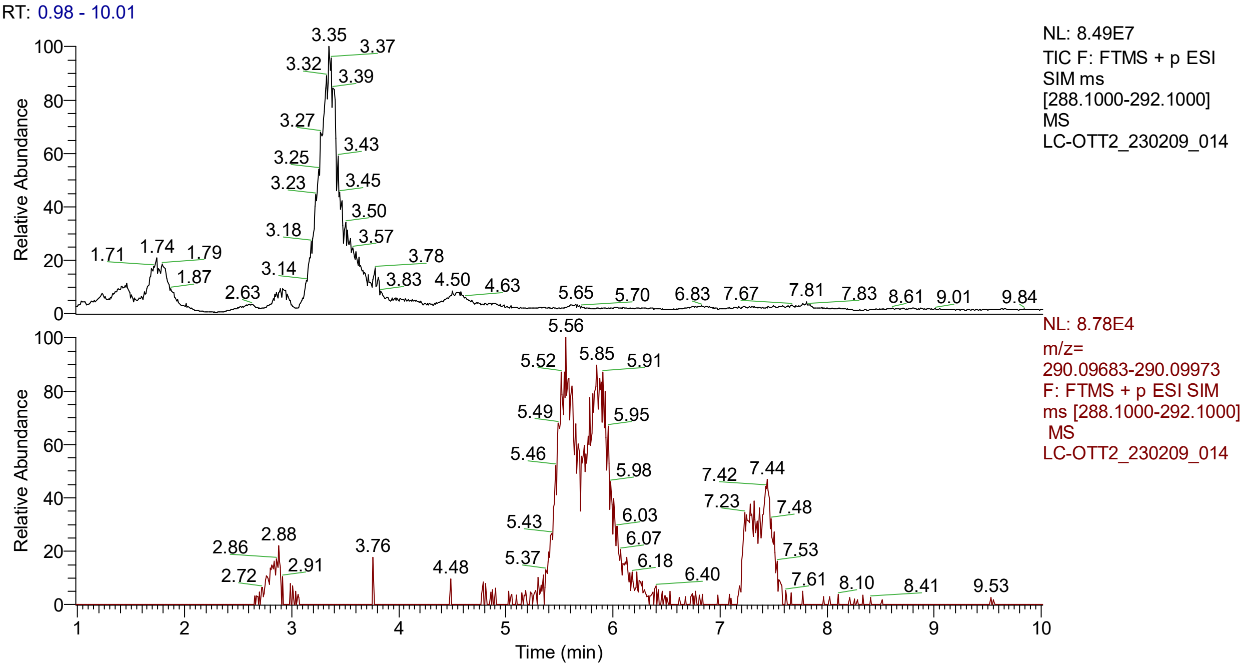

**Sample 3: SLV146 V37 09 / 09-19 OMR 164**

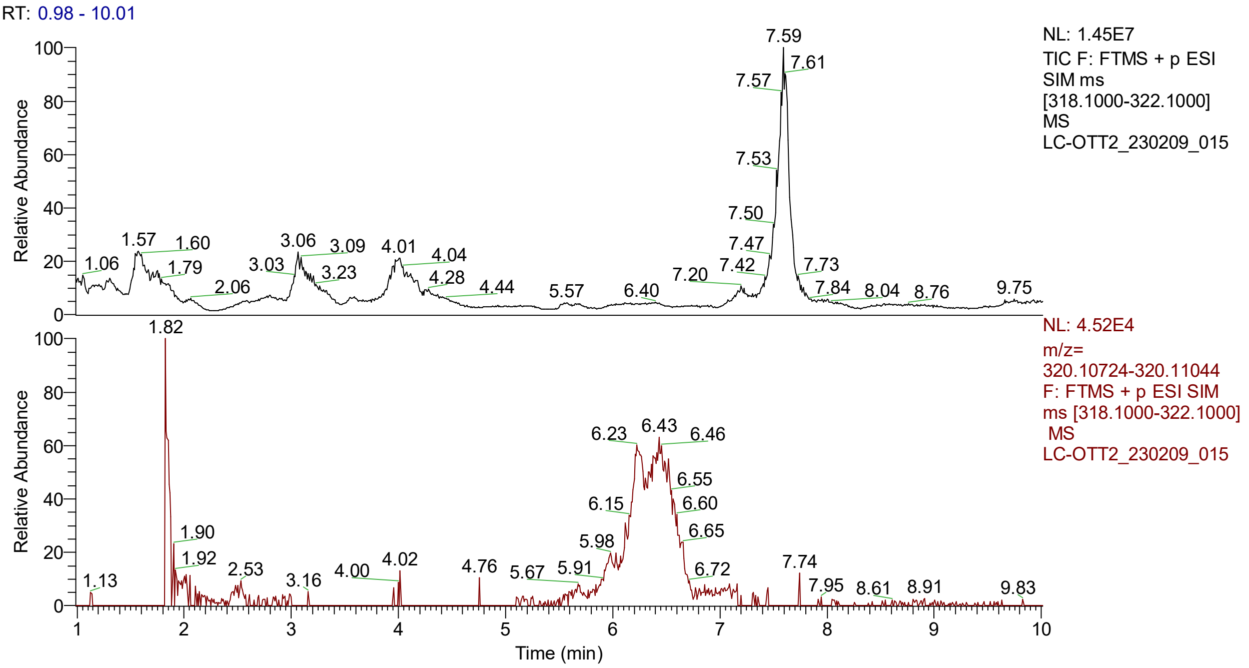


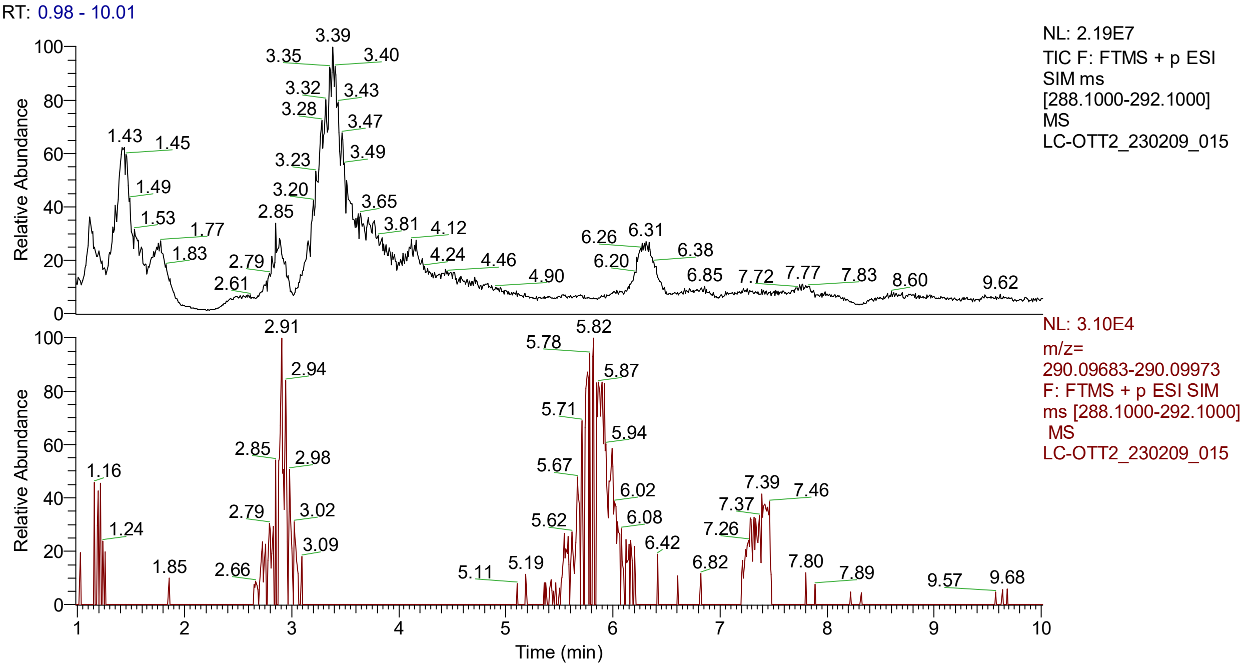

**Standard 2: 125 µg/kg TTX at 6.23 min.**

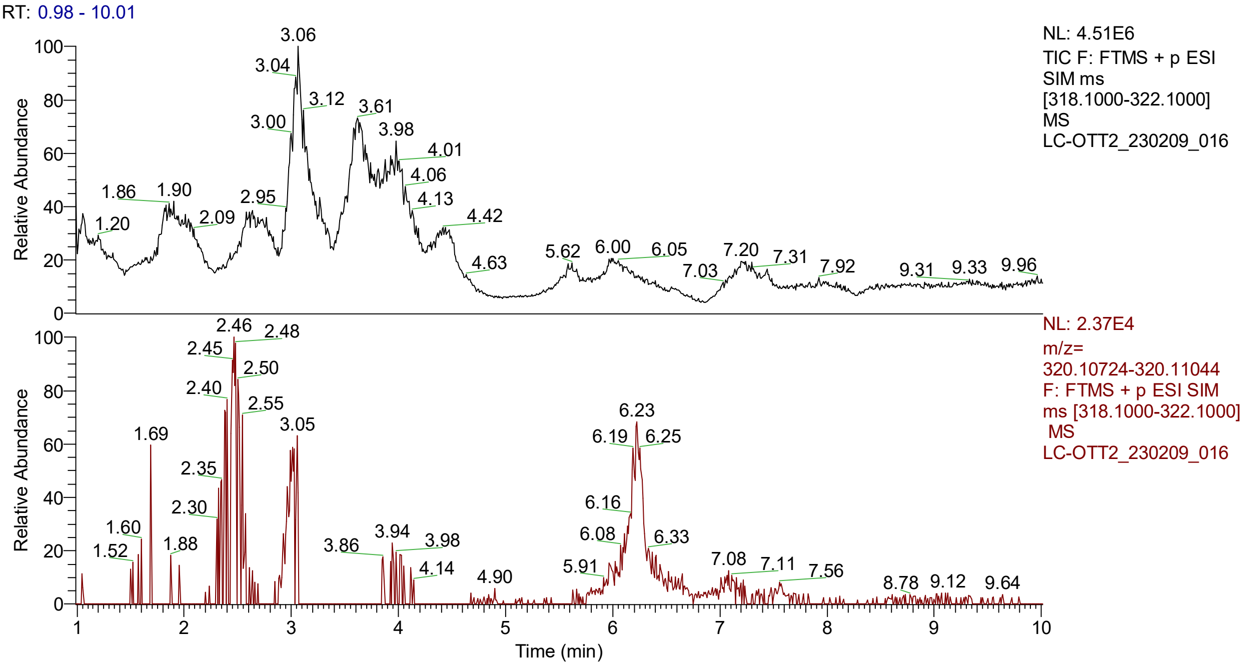


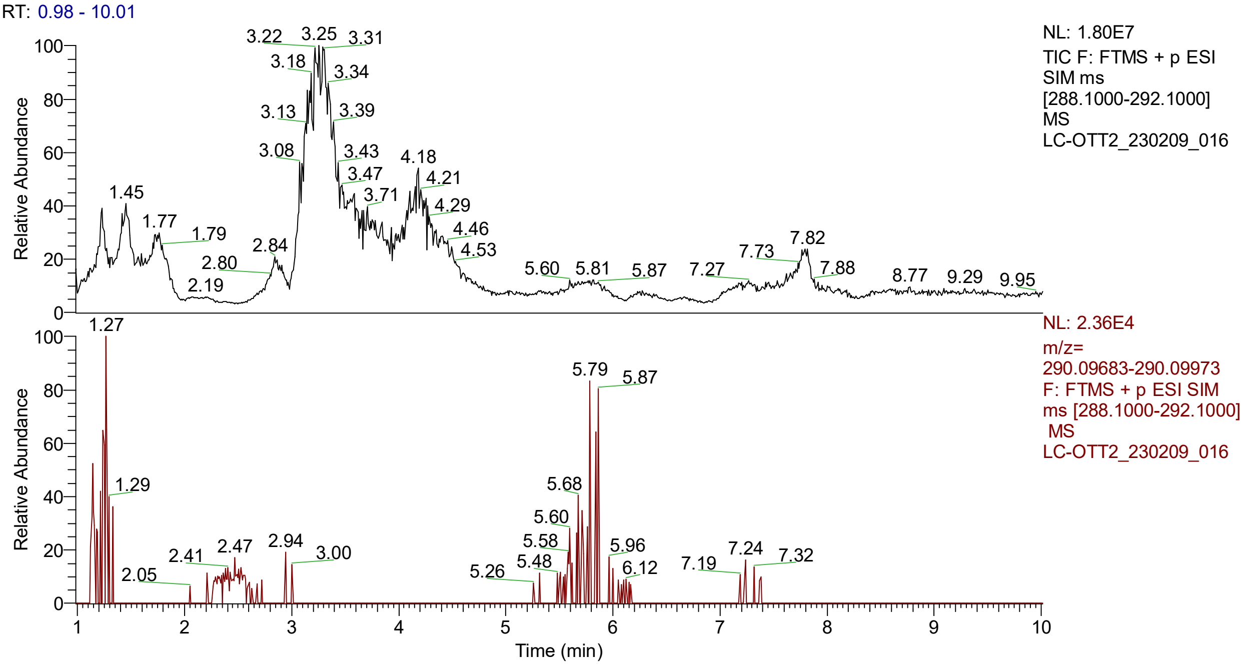

**Spectrum Standard 1**

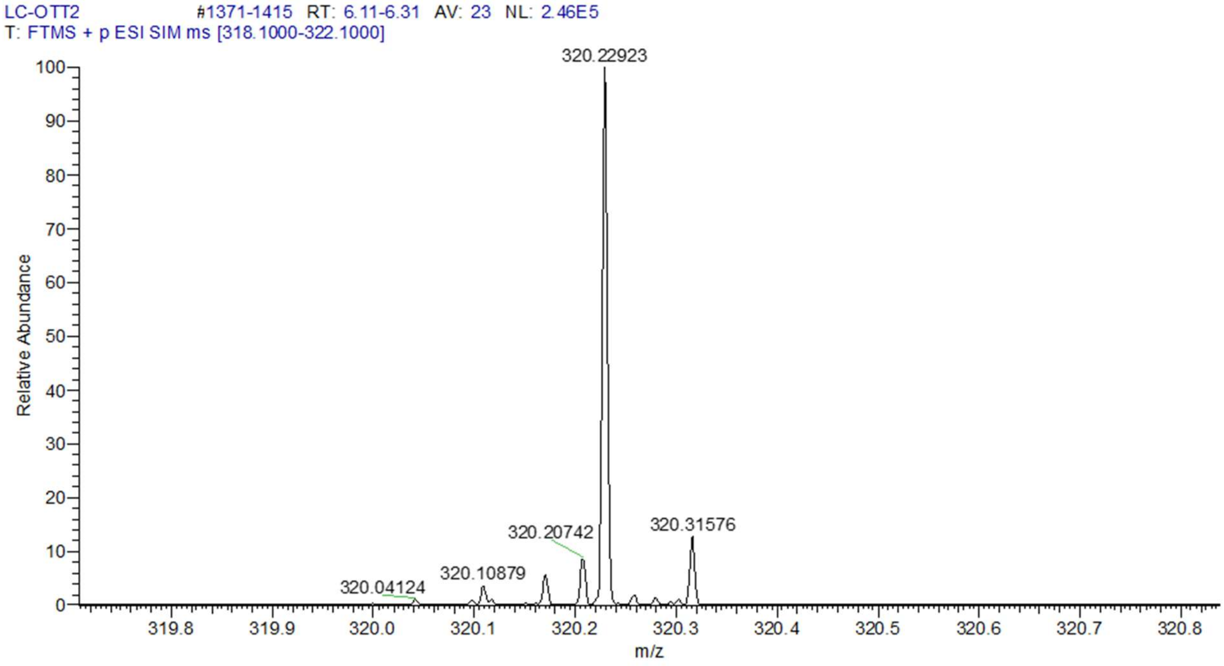


320.10879
ppm error -0.17

**Spectrum sample 1: SLV 062 V16 04 / 15-19 OMR 110**

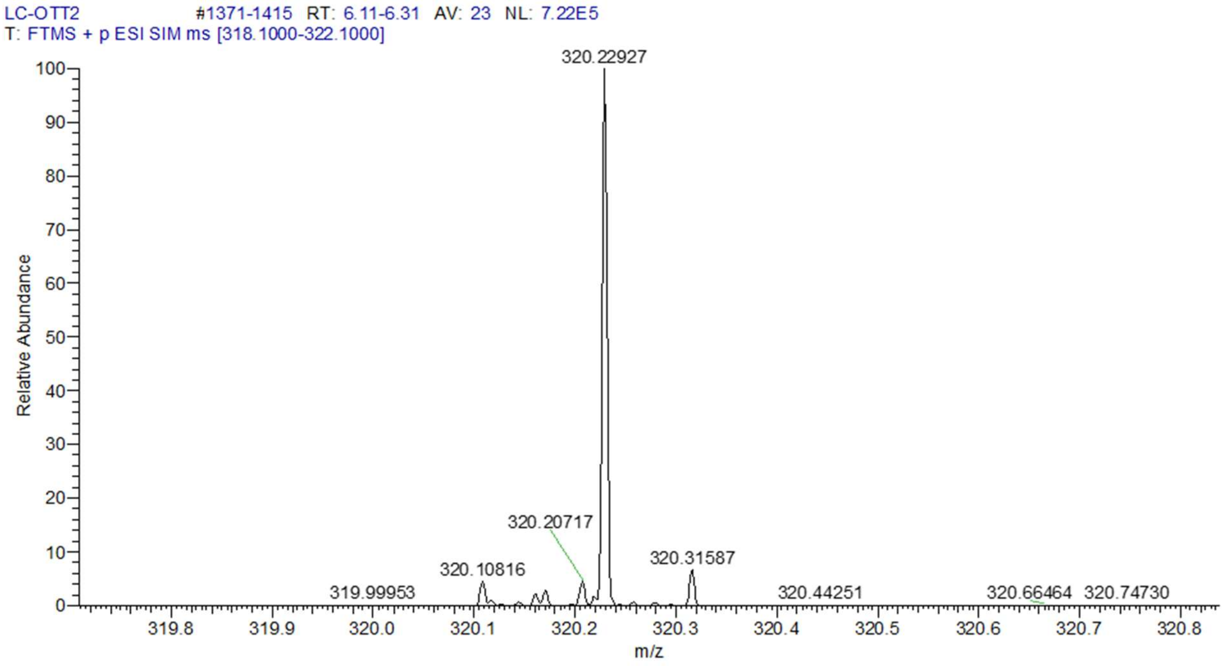


320.10816
ppm error -2.13

**Spectrum sample 2: SLV079 V20 05 / 11-19 OMR 110**

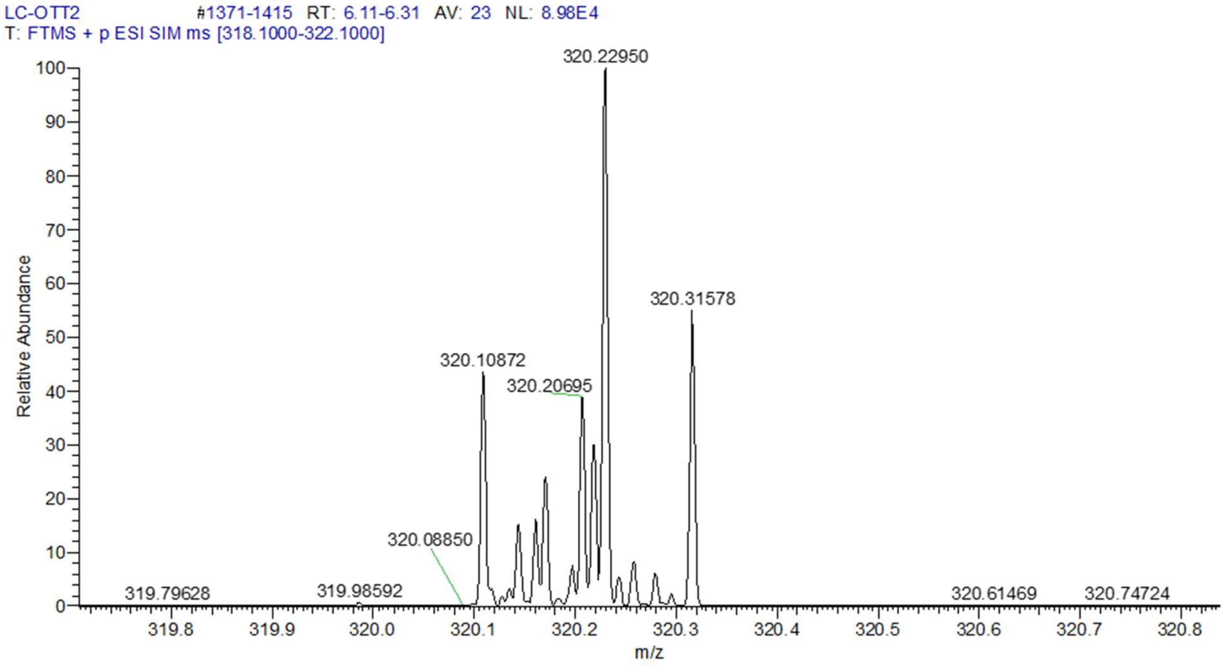


320.10872
ppm error -0.38

**Peak 5.60 min**

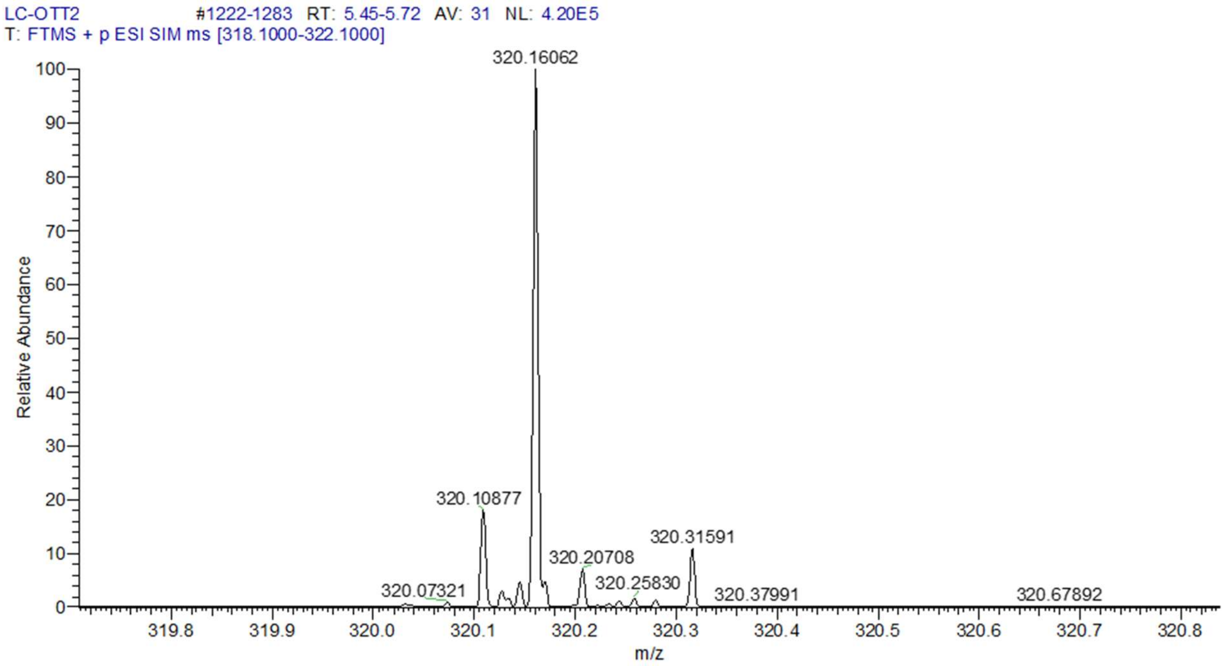


320.10877
ppm error -0.23

**Peak 5.60 min Fragmentation (retention time shift to 7.31 due to different analysis date)**

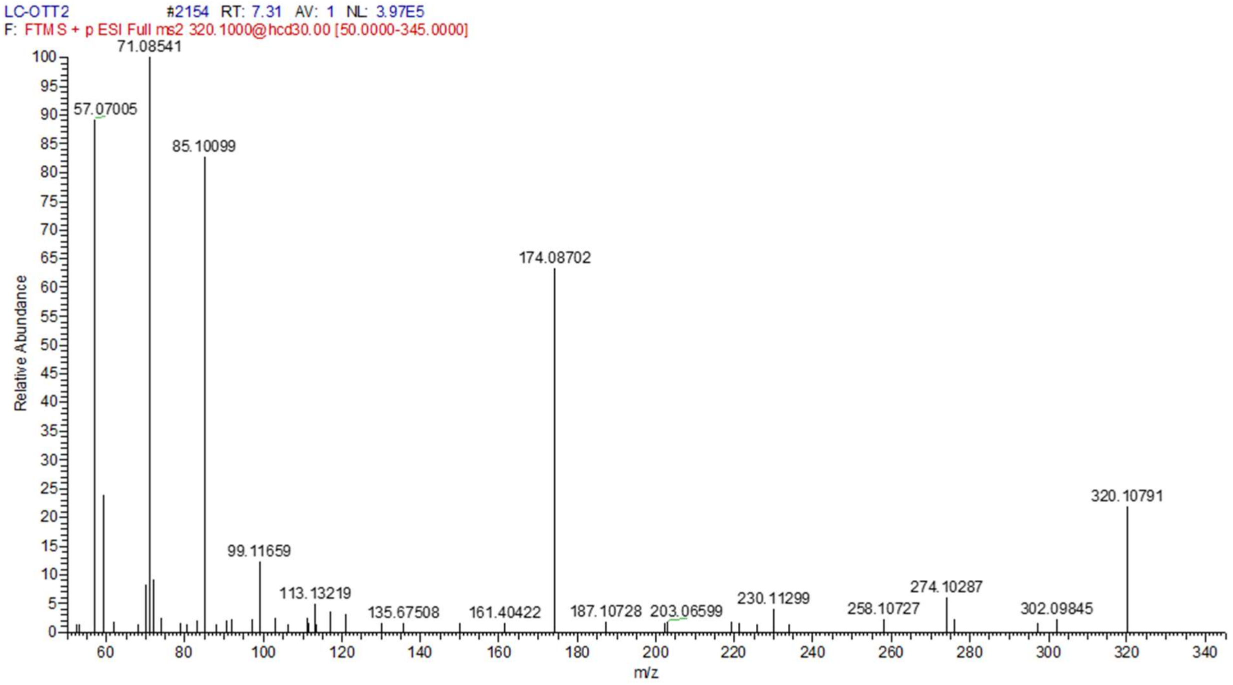


Fragment 302.09845
ppm error 0.56
Fragment 162 N/A

**Peak 5.56 min**

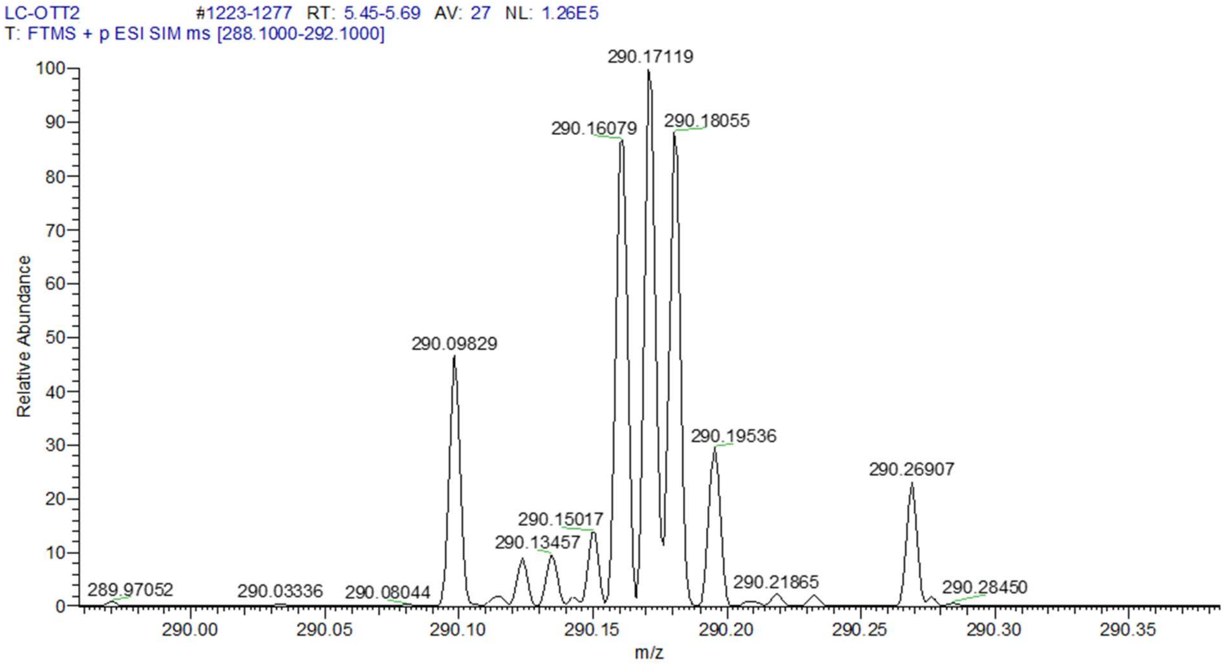


290.09829
ppm error 0.04

**Peak 5.85 min**

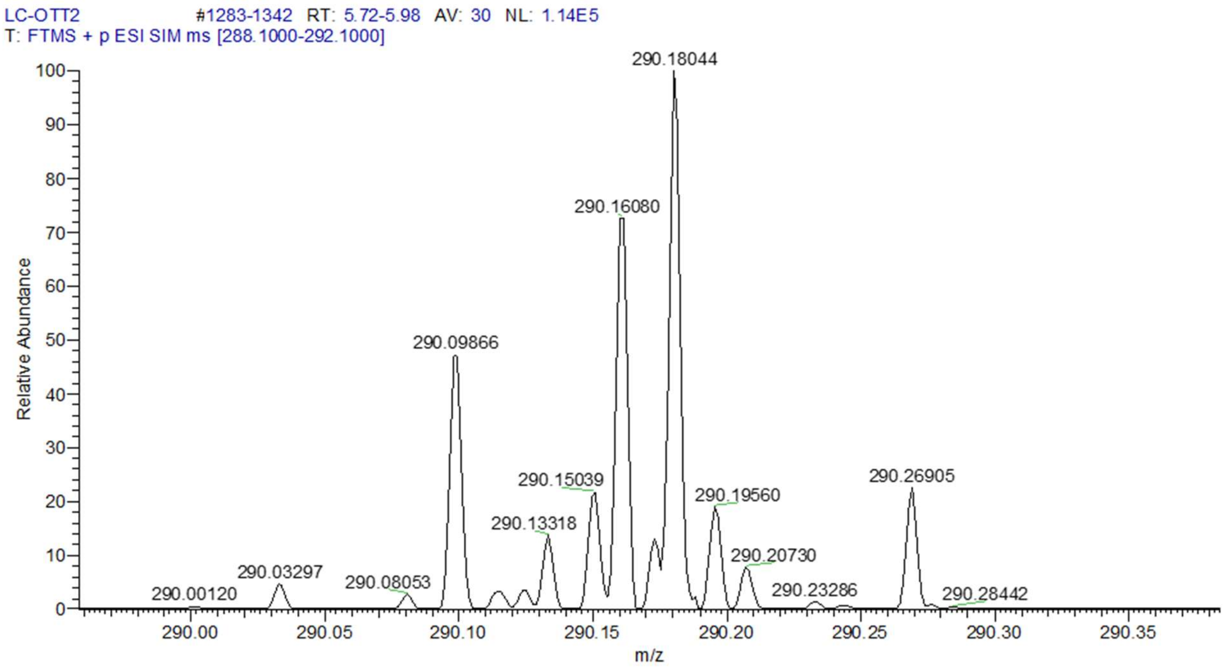


290.09866
ppm error 1.43

**Peak 7.45 min**

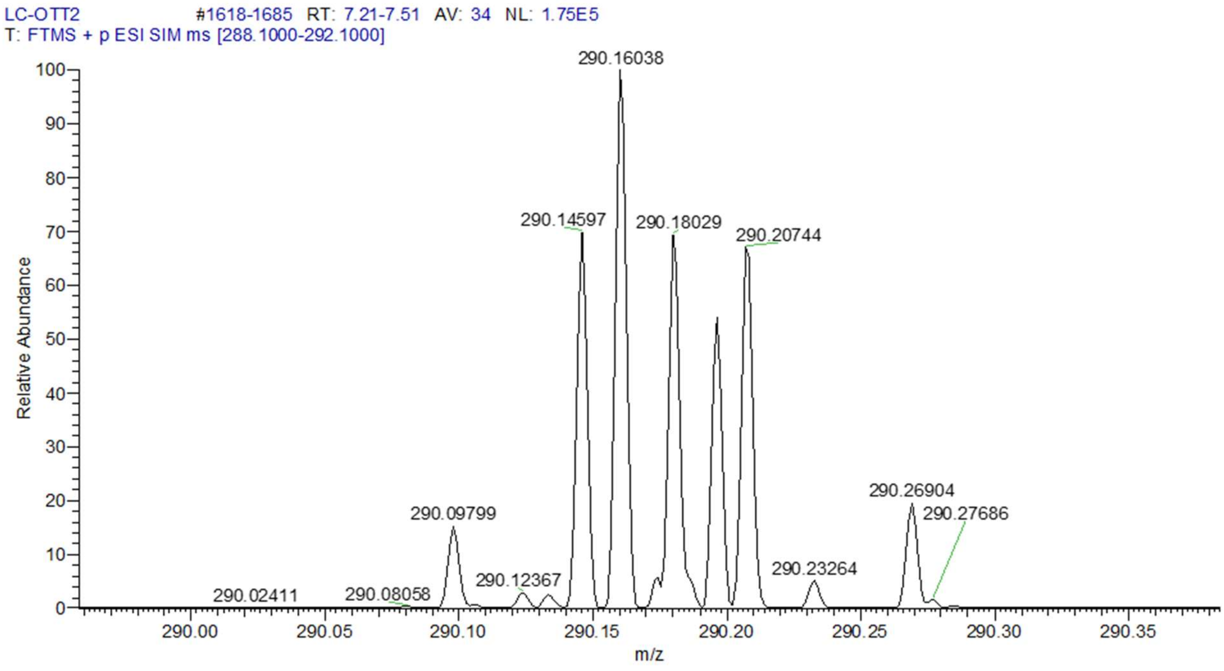


290.09799
ppm error -0.99

**Spectrum sample 3: SLV146 V37 09 / 09-19 OMR 164**

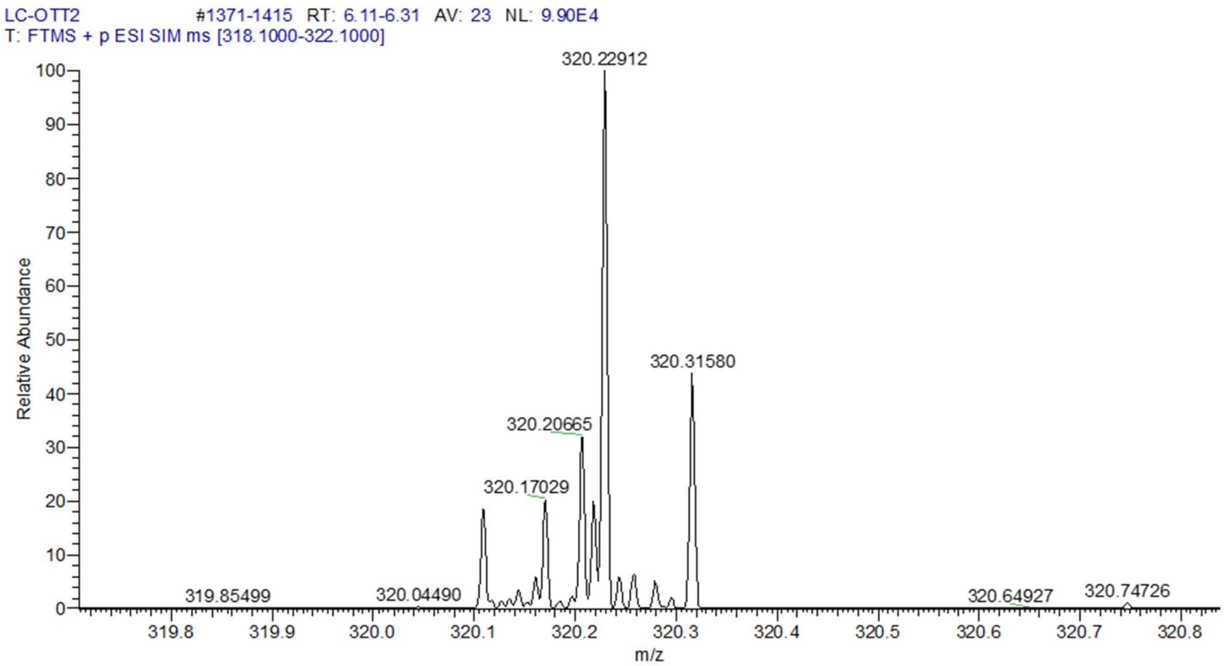


320.10886
ppm error 0.05

**Spectrum Standard 2**

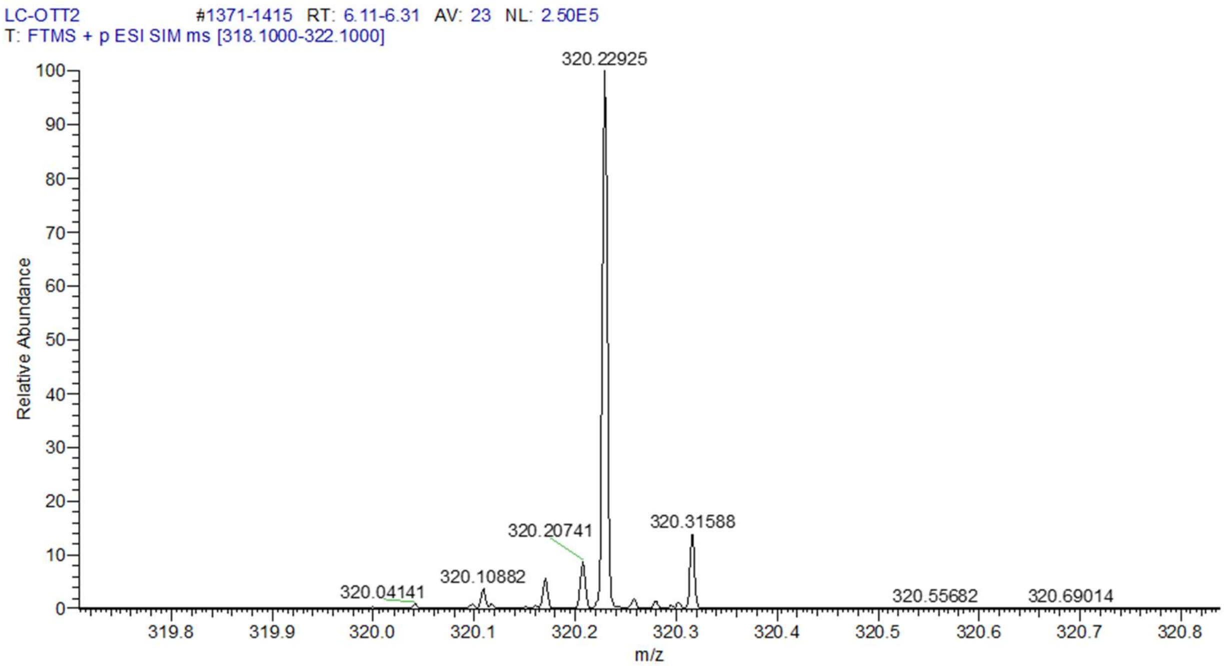


320.10882
ppm error -0.07
